# An Energy-Efficient MAC Protocol for Medical Emergency Monitoring Body Sensor Networks

**DOI:** 10.3390/s16030385

**Published:** 2016-03-17

**Authors:** Chongqing Zhang, Yinglong Wang, Yongquan Liang, Minglei Shu, Changfang Chen

**Affiliations:** 1 College of Information Science and Engineering, Shandong University of Science and Technology, Qingdao 266590, China; lyq@sdust.edu.cn; 2 Shandong Provincial Key Laboratory of Computer Networks, Shandong Computer Science Center (National Supercomputer Center in Jinan), Jinan 250101, China; wangyl@sdas.org (Y.W.); shuml@sdas.org (M.S.); chenchf@sdas.org (C.C.)

**Keywords:** body sensor networks (BSNs), energy efficient, medical emergency monitoring, MAC protocol

## Abstract

Medical emergency monitoring body sensor networks (BSNs) monitor the occurrence of medical emergencies and are helpful for the daily care of the elderly and chronically ill people. Such BSNs are characterized by rare traffic when there is no emergency occurring, high real-time and reliable requirements of emergency data and demand for a fast wake-up mechanism for waking up all nodes when an emergency happens. A beacon-enabled MAC protocol is specially designed to meet the demands of medical emergency monitoring BSNs. The rarity of traffic is exploited to improve energy efficiency. By adopting a long superframe structure to avoid unnecessary beacons and allocating most of the superframe to be inactive periods, the duty cycle is reduced to an extremely low level to save energy. Short active time slots are interposed into the superframe and shared by all of the nodes to deliver the emergency data in a low-delay and reliable way to meet the real-time and reliable requirements. The interposition slots can also be used by the coordinator to broadcast network demands to wake-up all nodes in a low-delay and energy-efficient way. Experiments display that the proposed MAC protocol works well in BSNs with low emergency data traffic.

## 1. Introduction

The development of body sensor networks (BSNs) has been boosted by the advances in wireless communication technology, sensors and batteries, miniaturization of electronics, *etc*. BSNs are also known as wireless body area networks (WBANs). With many prominent merits, BSNs are promising to be used in a wide range of application fields. BSNs are hoped to revolutionize the healthcare model by providing a new ambulatory treatment and monitoring approach, which only incurs little obstruction to the activities of patients and, thus, can emancipate the patients significantly [1].

Medical emergency monitoring is a typical type of BSN application. The difference between a common medical monitoring BSN and a medical emergency monitoring BSN lies in the data gathering mode. For a common medical monitoring BSN, the nodes sense the objects with a frequency and send all of the measurement data to the coordinator. Such a monitoring mode can gather full and accurate information about a patient, so as to let the medical staff understand the patient’s health condition precisely. The main drawback of this monitoring mode is the high energy consumption spent on transmitting the data. Different from a common medical monitoring BSN, the nodes in a medical emergency monitoring BSN do not send all of the data to the coordinator. On the contrary, a node sends the data to the coordinator only when it detects an emergency event [2].

Generally speaking, a medical emergency monitoring BSN adopts a star topology structure composed of one coordinator and a small number of sensor or actuator nodes. The coordinator coordinates the communications and collects the data from the nodes. Like traditional WSNs, energy efficiency is generally the most important goal when designing or deploying BSNs. For one thing, BSN nodes, especially the implanted nodes, are severely energy-constrained, because it is inconvenient or infeasible to replace or recharge the batteries that power the nodes. At the same time, these BSNs are expected to work for a considerable length of time, e.g., several months to even several years. This makes energy management become an apparent need for severely energy-constrained nodes. Without efficient energy management, a node may run out of its energy in only a couple of days. For a sensor node, the radio is generally the part that consumes the most energy. In order to extend the lifetime of a node from days to years, many researchers have focused their attention on how to design efficient methods to reduce the energy consumed by radios. To achieve this two orders of magnitude reduction in energy consumption, the only way for a node is duty cycling its radio, that is turning on its radio only when necessary and keeping it turned off otherwise [3].

To duty cycle the radio, a BSN node needs to sense the target with a certain frequency, yet the data are only transmitted to the coordinator on some occasions, e.g., when certain medical emergencies occur or the data are beyond predefined bounds, *etc.* [4]. The coordinator then informs the related medical staff and the patient’s family so that they can take timely measures to deal with the emergencies. BSNs can be used to monitor many sudden medical or non-medical emergent events, e.g., acute myocardial infarction, cerebral apoplexy, body falls, sudden changes of blood glucose, *etc*.

Medical emergency monitoring BSNs are helpful for the daily care of the elderly and chronically ill people, such as cardiovascular and cerebrovascular patients who are vulnerable to be attacked by myocardial infarction and cerebral apoplexy. For most of the time, there are no events occurring in a medical emergency monitoring BSN, and there is scarcely any traffic generated under these circumstances. To save energy, the radios of nodes should stay in sleeping mode as much as possible, that is to say the duty cycle when no event happens should be as low as possible. Yet, when an emergency happens, e.g., a myocardial infarction or a body fall occurs, the event should be reliably reported as soon as possible. Moreover, the nodes need to take a close look at the emergency and report it more frequently, so that the medical staff can closely track the situation of the patient. The traffic may burst to being very busy in a short time. Therefore, the traffic status when there is an emergency occurring is quite different from the traffic status when there is no event occurring. Accommodating these different traffic conditions is just the task of the medium access control (MAC) layer, which drives the radio hardware [5].

Different MAC schemes are needed to meet the demands of the different traffic statuses mentioned above. When there is no emergency occurring, the radios of nodes should be put into sleeping mode as much as possible by the MAC protocol to save energy. Energy efficiency is the primary goal on this condition. At the same time, the MAC protocol should provide mechanisms to ensure the real-time and reliability requirements of emergency data and awaken the network to waking mode when an emergency is detected. The duty cycle can be high in order to transport the emergency information in detail. Generally speaking, detailed information, not energy efficiency, is the primary goal in the case of an emergency.

Some existing MAC protocols, e.g., IEEE 802.15.4 and IEEE 802.15.6, can be adopted to meet the traffic demands in the case of an emergency. Yet, these protocols are not suitable for sleeping medical emergency monitoring BSNs, because they are not specially designed for such applications. For one thing, there is still scope to improve the energy efficiencies of these protocols. Secondly, these protocols lack an appropriate mechanism to immediately awaken the network. By this means, when certain emergencies, e.g., a body fall or cerebral apoplexy, are detected or the medical staff wants to obtain detailed information of the patient, the coordinator can awaken all of the nodes by issuing a wake-up command.

In this paper, a beacon-enabled MAC protocol is specially designed to meet the demands of sleeping medical emergency monitoring BSNs. The rarity of traffic in sleeping mode is taken into account to design the MAC protocol. A long superframe structure is adopted to avoid unnecessary beacons. By allocating most of the superframe to be inactive periods, the duty cycle is reduced to an extremely low level. Short active time slots are inserted into the superframe and shared by all of the nodes to deliver the emergency data in a low-delay and reliable way. These slots can also be used to deliver network demands and provide the coordinator one way to terminate the on-going superframe and begin a new one. Due to the rarity of traffic, the collision probability is very low. When there are more data need to be delivered and collisions happen, extra contention access period (CAP) periods are activated to transmit these data. Experiments show that the proposed MAC protocol can improve the energy efficiency and meet the real-time and reliability demands simultaneously in a BSN with low emergency data traffic.

Related works are introduced in the next section. Section 3 gives the design challenges and goals and introduces how the proposed MAC protocol works. A numerical performance analysis of the proposed protocol is presented in Section 4. Section 5 evaluates the performances of the proposed protocol. Finally, we draw conclusions in Section 6.

## 2. Related Works

A BSN belongs to a kind of sensor network for special purposes, such as medical applications, entertainment applications, sports training applications, *etc*. For traditional WSNs, there have been many MAC protocols. In [6], the authors have made a comprehensive survey about the MAC protocols for WSNs. They divided the WSN MAC protocols into four categories and studied the evolution of these protocols. B-MAC [7], PW-MAC [8], S-MAC [9] and DW-MAC [10] are some representative WSN MAC protocols. However, BSNs have specific characteristics in network structure, traffic pattern, network dynamics, *etc*. These characteristics make the MAC protocols for WSNs maladaptive to BSN applications.

IEEE 802.15.4 is the standard that defines the protocol and interconnection operations for devices in a wireless personal area network (WPAN). IEEE 802.15.4 can also be customized or revised to accommodate BSN applications, e.g., healthcare and consumer electronics applications [11]. IEEE 802.15.4 has two communication modes: beacon mode and non-beacon mode. Each mode has its advantages and disadvantages. To cater to diverse applications of BSNs, IEEE Task Group TG6 released 802.15.6 as the standards for BSNs. IEEE 802.15.6 is designed to be a versatile communication suite, which is flexible enough to adapt to many kinds of applications. However, this adaption is not accomplished automatically by IEEE 802.15.6 itself. In fact, 802.15.6 only defines the basic elements that ensure the interoperability among BSN devices, and it leaves the questions of customization and adaption to application developers. In this sense, 802.15.6 is not a complete MAC protocol [12].

Besides the two aforementioned industry standards, there are other BSN MAC protocols [13,14,15,16,17,18,19] designed by the academic or industrial community. However, these protocols are designed for general applications with heavy traffic. There are also several MAC protocols [20,21] specially designed for handling emergency events in BSNs. However, these protocols did not take the rarity of traffic into consideration to design an effective mechanism to improve the energy efficiency. In [22], an MAC protocol was proposed by Shu *et al*. to deliver urgent data for BSNs with heavy traffic. Yet, this protocol is not specially designed for medical emergency monitoring applications and does not consider the specific characteristics of such applications. As a subsequent work of [22], this paper redesigns the MAC mechanism of [22] to adapt to medical emergency monitoring BSN applications.

## 3. MAC Protocol Design

### 3.1. Design Challenges and Goals

To monitor medical emergencies, BSN nodes need to periodically monitor many physiological or physical phenomena or objects, e.g., body temperature, blood pressure, heart rate, acceleration, force, *etc*., and judge the measurements to determine if there are emergencies occurring. Once an emergency is detected, the data should be immediately reported to the coordinator in a reliable way. Because the occurrence probability of emergencies is low and random, the traffic generated by medical emergency monitoring is low and random. Depending on the patient’s health condition and the node’s data rate, the packet rate of a node may be one-hundredth pps (packets per second) or even lower, and the packet size may range from a few bytes to several kilobytes. For emergency data, the real-time and reliability demands are very high in general.

The proposed MAC protocol is designed for the above-mentioned medical emergency monitoring applications, and hence, we call this MAC protocol MEM-MAC (medical emergency monitoring MAC). For most of the time, there is no emergency occurring. MEM-MAC is just designed for monitoring the occurrence of emergencies in such a time. Such a network generally exhibits a star structure composed of one single coordinator and many other heterogeneous nodes, including sensors and actuators. The nodes are severely energy-constrained because they are powered with batteries, which cannot be recharged or replaced easily. Medical emergency monitoring applications are often used for the daily care of the elderly and chronically ill people. Although the coordinator can be easily equipped with a rechargeable battery, it is common for a senior person to forget to recharge the coordinator. This means the coordinator is also energy-constrained, and energy saving is also meaningful for the coordinator. A medical emergency monitoring BSN is generally expected to serve the patient as long as possible. This makes energy management an urgent need for severely energy-constrained nodes and the coordinator. As discussed above, the only way to extend the lifetime by leaps and bounds is duty cycling the radio, that is turning on the radio only when necessary and keeping it turned off otherwise.

In a medical emergency monitoring BSN, there are no events occurring for most of the time, and there is scarcely any traffic generated under these circumstances. This feature can be exploited to design energy-saving approaches for the nodes and the coordinator. To save energy, the radios of nodes should stay in sleeping mode as much as possible, that is to say, the duty cycle when no event happens should be as low as possible. However, the low duty cycle should not hamper the real-time and reliability demands of the medical emergency data. That is to say, an emergency event should be reliably reported to the coordinator as soon as possible regardless of what low duty cycle is adopted.

Moreover, it is necessary to provide a fast wake-up mechanism to awaken the sleeping nodes so as to rouse the BSN into waking mode to track the event closely. When an emergency happens, it is often accompanied by some accidental events or some abrupt changes of the patient’s health condition, which should be traced closely by the BSN. These occasions are just where the fast wake-up mechanism comes into play. Using such a mechanism, the BSN can be woken up quickly and promptly collect abundant information of the patient.

To cope with the above-mentioned challenges, this paper tries to design an MAC protocol for medical emergency monitoring BSNs. The proposed protocol tries to design effective energy-saving approaches for both the coordinator and the nodes, designs real-time and reliability communication methods for medical emergency events and offers a mechanism to awaken the BSN rapidly.

**Figure 1:**
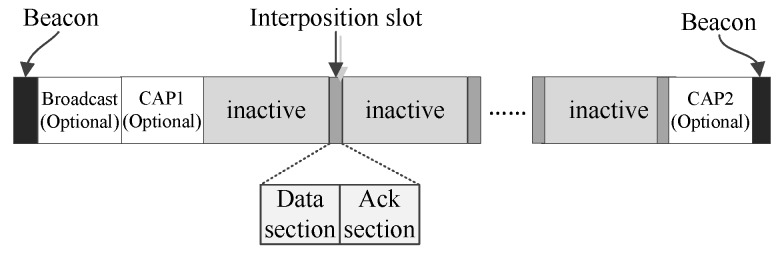
The structure of the medical emergency monitoring (MEM)-MAC superframe.

Figure 1 shows the superframe structure of MEM-MAC. The basic idea of MEM-MAC is interposing short active time slots into the long inactive period and using these time slots to convey the emergency data and network commands. To some extent, these time slots can be deemed as the combination of beacon frames and active periods of traditional BSN MAC protocols, e.g., IEEE 802.15.4 and 802.15.6. In this paper, these interposed time slots are called interposition time slots. To convey data using interposition slots, MEM-MAC divides data into two classes: small data and big data. A data that can be directly transmitted by one interposition slot is a small data. A data with a bigger size and cannot be directly transmitted by one interposition slot is a big data. How to convey a big data will be introduced later. MEM-MAC has the following features:

(1) High energy efficiency for both the coordinator and the nodes: To transmit the emergency data with high real-time demands, existing MAC protocols generally employ a short superframe, which means short beacon frame intervals. This incurs plenty of unnecessary beacon frames that cause unnecessary energy consumption. To save the energy wasted on beacon frames and guarantee the real-time demands, MEM-MAC adopts long superframes and short interposition time slots. On the other hand, the proposed MAC protocol does not need to let the coordinator keep on listening all the time. This reduces the duty cycle of the coordinator and improves its energy efficiency.
(2) Real-time and reliable communication: Interposition time slots are adopted by the MEM-MAC to convey medical emergency data. The intervals between interposition time slots can be adjusted according to the occurrence frequency of emergency data, so as to satisfy the real-time demands. When big emergency data with high priority are generated, the currently on-going superframe will be terminated, and the coordinator will begin a new superframe in which a CAP1 period is activated to transmit the big emergency data. For emergency data with a high reliability demand, the MEM-MAC protocol adopts acknowledgment and retransmission frames to guarantee the successful transmission of frames. To do this, an interposition time slot is partitioned into two sections, the data section and the ack section. The data section is used to transmit emergency data or network commands, and the ack section is used to acknowledge the transmission.
(3) Rapid wake-up mechanism: A wake-up mechanism is actually a broadcast mechanism. Using MEM-MAC, both the data section of a download interposition slot and the ack section of an upload interposition slot can be used by the coordinator to broadcast a wake-up message to awaken all nodes into waking mode. Besides, the coordinator can also use an interposition slot to terminate the on-going superframe and begin a new superframe with a broadcast period to broadcast messages to all nodes.

### 3.2. Structure of the Superframe

It can be seen from Figure 1 that the superframe of MEM-MAC has an adaptive structure. A superframe begins with a beacon frame, which contains control information. There may exist an optional broadcast period and an optional contention access period (CAP) following the beacon. The optional broadcast period is used by the coordinator to broadcast long frames to all of the nodes. The optional CAP1 period can be used by the coordinator to transmit a long frame to one node or can be used by one node to transmit a long frame to the coordinator. CSMA is adopted as the medium access mechanism in CAP1. The existence of these two optional periods depends on the foregoing superframe. If big data were generated by the coordinator during the forgoing superframe, the coordinator would terminate the foregoing superframe and start a new one. The optional broadcast period is activated in the new superframe and is used to broadcast the long frame containing the big data. If big emergency data were generated by one node during the previous superframe and the data generator has sent a request to the coordinator, then the optional CAP1 period is activated for the node to convey its data.

Following the CAP1 period is a long inactive period into which the interposition slots are inserted. Because there is no emergency occurring for most of the time, the inactive period occupies most of the superframe. The interposition slots are shared by all of the nodes to transmit data or network commands. The effectiveness of this mechanism is based on the rarity of traffic in sleeping mode, that is there are only rare emergency events occurring between two interposition slots. Hence, even though all nodes share one interposition slot, collisions seldom happen. On occasions that collisions happen frequently, the interposition slot mechanism does not work well.

Normally, a superframe ends with an inactive period. Sometimes, a superframe may end with an optional CAP2 period. The presence or absence of CAP2 relies on if there were collisions occurring. On occasions where there were frames colliding in one interposition slot, a CAP2 period is installed to transmit these frames again. CSMA is also adopted as the medium access mechanism in CAP2. After these, the superframe ends, and a new superframe starts.

MEM-MAC uses two kinds of interposition slots, upload interposition slots and download interposition slots, to support bidirectional communications. Upload interposition slots are designed to convey frames from the nodes to the coordinator, and download interposition slots are used to deliver frames from the coordinator to the nodes. One interposition slot comprises two sections, one data section and one ack section. For a download interposition slot, the coordinator is the data sender, and all nodes are data receivers during the data section. Conversely, the coordinator is the data receiver, and some nodes are data senders during the data section for an upload slot.

MEM-MAC uses data sections to deliver data frames bidirectionally. Data frames may encapsulate emergency data, network or uses commands, *etc*. Ack frames are transmitted in ack sections to acknowledge the data frames to satisfy the reliability requirements. MEM-MAC only acknowledges unicasts. Besides, MEM-MAC can also use ack sections to broadcast special network commands, like “BREAK”, “CAP” and “SYNC” to all nodes. A download interposition can play the same role. The “BREAK” command frame is used to break the on-going superframe and begin a new frame. The broadcast of a “CAP” command frame signifies a CAP2 period will begin after the ack section. The length of this period is included in the “CAP” command frame. After the CAP2 period, a new superframe begins. The “SYNC” command frame is designed to synchronize time between the coordinator and the nodes.

All intervals between two neighboring interposition slots in a superframe have a fixed length. Yet, the interposition intervals of different superframes may have different lengths. The interval between two adjacent interposition slots depends on the production of the emergency data and commands. For download interposition slots and upload interposition slots, things are different. The interval between two adjacent upload interposition slots is decided by the occurrence frequency of the emergencies and commands of all nodes, while the interval between two adjacent download interposition slots is decided by the occurrence of events and commands on the coordinator.

### 3.3. Operations of MEM-MAC

Because there are two types of interposition slots, the operations of MEM-MAC are correspondingly divided into two types, download operations and upload operations. We first introduce the case that there is no traffic generated during an interposition interval, and then, the download operations and upload operations are introduced respectively.

**Figure 2:**
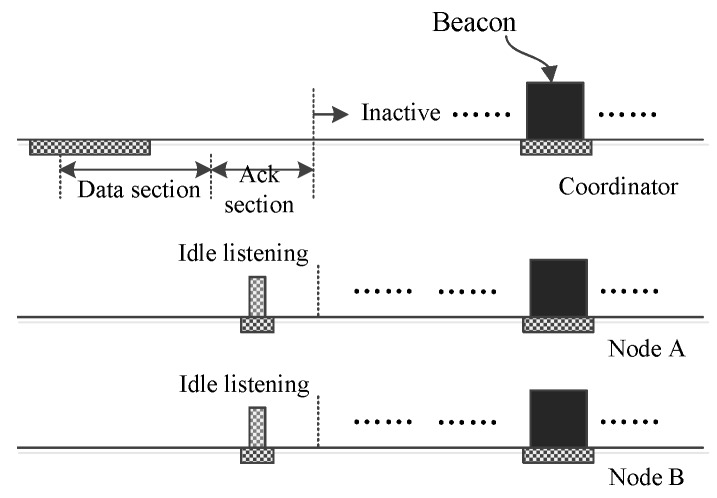
No traffic occurs during two adjacent interposition slots.

In an emergency monitoring BSN, it is usual that there is no traffic occurring during two adjacent interposition slots. Figure 2 shows the operation handling the case that there is no upload traffic occurring for an upload slot. As the figure shows, during the data section, all nodes reside in sleeping mode. Yet, the coordinator keeps on listening to the channel to receive potential frames from nodes. Once it realizes there is no traffic in the BSN, it abandons listening and turn off its radio to save energy. In the following ack section, all nodes need to wake up to receive a potential frame from the coordinator. If they find there is no such frame, they go back to sleep to save energy. The operation handling the case that there is no download traffic occurring is similar to Figure 2 and is not given further in this paper.

**Figure 3:**
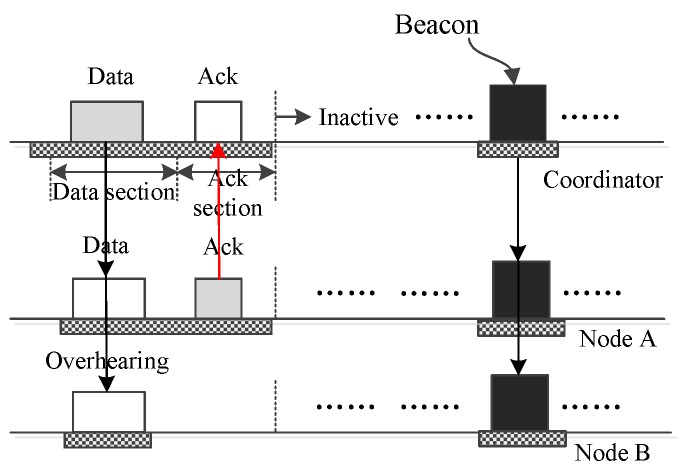
The coordinator sends small data to one node, A.

Figure 3 illustrates the operation in which the coordinator sends one small data frame to one node A. As in the figure, the data are encapsulated into a frame and sent out by the coordinator during the data section. All nodes listen to the channel to receive the frame. After the reception, only node A finds that the frame is sent to it, and other nodes, like node B, will drop the frame and shut off their antennae. In the following ack section, node A acknowledges the data frame. The operation in which the coordinator broadcasts a small data frame to all nodes is similar to the operation shown in Figure 3. The difference is there is no acknowledgment.

**Figure 4:**
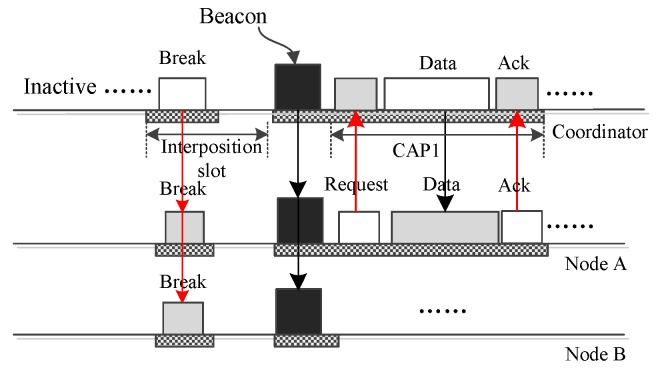
The coordinator sends big data to one node, A.

Figure 4 gives the operation in which the coordinator sends urgent big emergency data to a node A. Limited by the data frame size, big data cannot be encapsulated into an interposition data frame. To convey the big data, the coordinator needs to activate an extra period, *i.e.*, CAP1 period. To do this, the coordinator creates a “BREAK” frame with the broadcast address and broadcasts it using the data section of a download interposition slot. This “BREAK” frame can also be transmitted using the ack section of an upload slot. This “BREAK” frame terminates the on-going superframe and starts a new one in which the coordinator activates the CAP1 period. Then, during the CAP1 period, the node retrieves the data from the coordinator.

**Figure 5:**
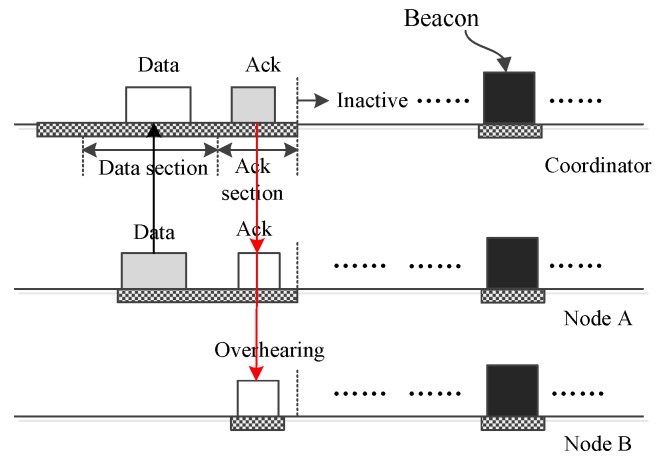
Only one node sends small data to the coordinator.

The operations of upload interposition slots are introduced in the following. The occasion in which only one node sends small data to the coordinator is first introduced. Figure 5 gives such an example of how node A transmits small data. As the figure shows, the data can be packed completely into an upload data frame and can be transmitted to the coordinator during the data section. After receiving the frame, the coordinator acknowledges the frame by broadcasting an ack frame. From the ack frame, node A knows the data frame has been received successfully. For other nodes that do not have data to send, they stay in sleeping mode in the data section. These nodes only need to wake up to receive the potential broadcast frame from the coordinator. The operation of such a node is illustrated by node B in Figure 5. Once node B perceives that the ack frame is not sent to it, node B discards the frame and goes back to sleep.

**Figure 6:**
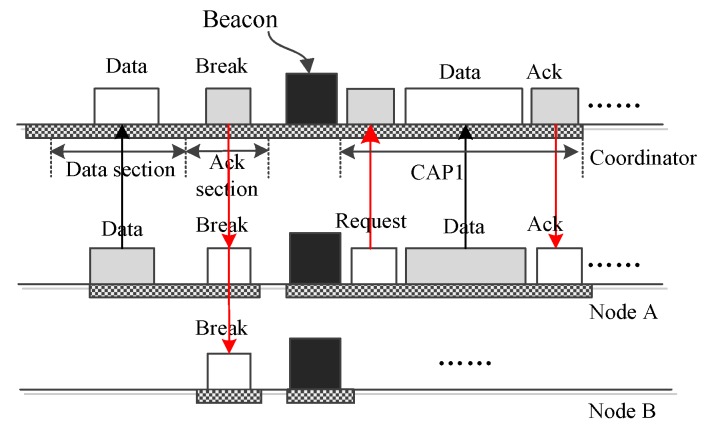
Only one node sends big emergency data to the coordinator.

Figure 6 shows how MEM-MAC conveys big emergency data from one node to the coordinator. If node A has big emergency data to transmit, it sends a request frame containing the data size to the coordinator. Upon accepting the request, the coordinator breaks the current superframe with a “BREAK” frame. This “BREAK” frame terminates the on-going superframe and begins a new one in which the CAP1 period is activated. Node A will use the CAP1 period to transmit the big emergency data to the coordinator.

**Figure 7:**
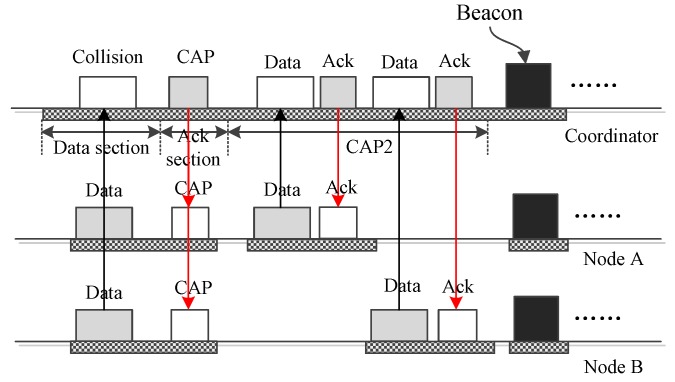
Two nodes send data to the coordinator.

There may be two or more nodes that want to send data during one interposition slot. On such an occasion, simultaneous transmissions of these frames will lead to the occurrence of collisions. In the case shown by Figure 7, nodes A and B have data to send in the same data section. This causes a collision and two communication failures. The coordinator cannot recognize the frames, and then, it broadcasts a “CAP” frame, including a field indicating the length of the CAP period. This “CAP” frame activates a CAP2 period for the nodes to compete to transmit their data. The length of the CAP2 period is set according to the history, *i.e.*, the data occurrence frequency calculated by the coordinator. In practice, a longer value should be set to accommodate more potential frames. If one datum is small data, then the data will be transmitted directly by the node. If one datum is big data, then a request will be transmitted. The actual transmission of the big data goes in the CAP1 period of the new superframe. After the CAP2 period, all nodes wake up to receive a new beacon frame, then begin a new superframe.

Time synchronization is important to beacon enabled MAC protocols. Because a long superframe is used by MEM-MAC, the incurred long clock drifts make the time synchronization problem severer for this protocol. Out-syncs may lead to energy waste and communication failures. To make up the long clock drifts caused by the long superframe, besides beacon frames, MEM-MAC uses interposition slots to send time synchronization information. The information is transmitted by “SYNC” frames containing the number of elapsed time slots from the beginning of the superframe. The ack section of an upload interposition slot and the data section of a download interposition slot can both be used.

Beacon frames, “CAP”, “BREAK” and “SYNC” frames are all broadcast by the coordinator to all nodes. However, the successful reception of a broadcast frame by all nodes cannot be assured. In a network in which the channel condition is good, failing to receive broadcast frames does not happen frequently. Yet, in a network in which the channel condition is bad or the spectrum is crowded, the broadcast failure may happen frequently. Failing to receive a broadcast frame may cause the loss of time synchronization or even the disassociation of a node. A node that loses association with the coordinator is called an orphan. One orphan who wants to join the BSN has to listen to the channel for beacon frames and starts an association process by sending an “ASSOCIATE” frame to the coordinator. On the contrary, a node who wants to leave the BSN can send a “DISASSOCIATE” frame to the coordinator to start a disassociation process.

The arrangements of the upload and download slots in a superframe is defined in the beacon frame. The beacon frame defines the first places of the first upload and download slots, and it also defines the two intervals for the upload slots and download slots. The interval between two slots is fixed, and the interval is calculated based on the occurrence frequency of emergencies. Because the number of nodes and upload events outdistance the number of coordinator and download events, the download slot interval should be much longer than the upload slot interval. In the implementation, this is handled by inserting one download slot every certain number of upload slots.

## 4. Performance Analysis

The analytical approach used in [23,24] is adopted in this section to analyze the energy efficiency and delivery latency of MEM-MAC. A body sensor network can be represented as $BSN=\left\{C,N_{1},N_{2},\ldots ,N_{n}\right\}$, in which *C* is the coordinator and $\left\{N_{1},N_{2},\ldots ,N_{n}\right\}$ denotes the nodes set. The emergency events on all nodes are assumed to occur according to the Poisson process, and all of the processes are assumed to be independent. Let $E_{B}=\left\{\lambda_{1B},\lambda_{2B},\ldots ,\lambda_{nB}\right\}$ be the set of the average big emergency data arrival rates of all nodes, and let $E_{S}=\left\{\lambda_{1S},\lambda_{2S},\ldots ,\lambda_{nS}\right\}$ be the set of the average small emergency data arrival rates of all nodes. Based on the random process theory, the above emergency data arrival rates sets $E_{B}$ and $E_{S}$ can be merged to be $E=\{\lambda_{1},\lambda_{2},\ldots ,\lambda_{n}\}$, where $\lambda_{i}=\lambda_{iB}+\lambda_{iS}$. All big emergency data arrival processes can be merged to be a new process with arrival rate $\lambda_{B}=\sum_{i=1}^{n}\lambda_{iB}$. Similarly, all small emergency data arrival processes and all of the emergency data arrival processes can form two new process with arrival rate $\lambda_{S}=\sum_{i=1}^{n}\lambda_{iS}$ and $\lambda =\lambda_{B}+\lambda_{S}$.

Because MEM-MAC operates under beacon mode, the duty cycle contributed by the periodical beacon frames needs to be first calculated. The averaged duty cycle incurred by receiving beacons for a receiver can be expressed as [23]:

(1) $$DC_{BF}=\frac{2\left(\varepsilon_{\text{TX}}+\varepsilon_{RX}\right)\cdot BI+T_{Beacon}+T_{TX\_wu}+T_{RX\_wu}}{BI}$$

In Equation (1), $2\left(\varepsilon_{\text{TX}}+\varepsilon_{RX}\right)\cdot BI$ denotes the sum of the clock drifts of the transmitter and receiver between BI, which means the beacon interval; the beacon frame transmission time is $T_{BF}$; $T_{TX\_wu}$ and $T_{RX\_wu}$ denote the wake-up times of the transmitter and receiver, respectively.

In a BSN that adopts MEM-MAC protocol, the emergency data are transmitted using the operations described in Section 3. Next, we calculate the energy consumed for a node to receive frames. For the upload operations, the receiving operations include receiving beacons, receiving acknowledgment frames or broadcast frames during the ack section. For the download operations, the receiving operations include receiving data and potential adaptive beacons. To sum up, the average power consumed for receiving operations can be written as:

(2) PNode_RX=(DCBF+Ti_ack+TRX_wuTEvent_up+Ti_data+TRX_wuTEvent_down+Ti_ack2+2εTX+εRX·SI+TRX_wu+Px=1,S·Ti_ack/2n+(Px=1,B+Px≥2)(Ti_ack2+TBeacon+TRX_wu)Tint_up+Ti_data2+2εTX+εRX·SI+TRX_wu+Py=1,S·Ti_data/2n+(Py=1,B+Py≥2)(Ti_data2+TBeacon+TRX_wu)Tint_down)PRX

where $DC_{BF}$ signifies the duty cycle contributed by the regular beacons, $\frac{T_{i\_ack}+T_{RX\_wu}}{T_{Event\_up}}$ denotes the duty cycle consumed by the acknowledgment frames for upload emergency data and $\frac{T_{i\_data}+T_{RX\_wu}}{T_{Event\_down}}$ denotes the duty cycle spent by the download data. $T_{Event\_up}$ and $T_{Event\_down}$ signify the average upload and download data occurrence intervals. The rest of the energy is consumed for the nodes to receive adaptive beacons, potential broadcast frames and download data from the coordinator. $I_{Int\_up}$ and $I_{Int\_down}$ represent the upload and download interposition slot intervals. $2\left(\varepsilon_{\text{TX}}+\varepsilon_{RX}\right)\cdot SI$ denotes the sum of the clock drifts of the transmitter and receiver between SI, which denotes the interval between two “SYNC” frames. $P_{x=1,S}$, $P_{x=1,B}$ and $P_{x\geq 2}$ denote the probabilities that only one small upload datum, only one big upload datum and more than one upload datum were generated during the one upload interposition interval. $P_{y=1,S}$, $P_{y=1,B}$ and $P_{y\geq 2}$ signify the probabilities that only one small download datum, only one big download datum and more than one download datum were generated during one download interposition interval. $P_{x=1,S}$, $P_{x=1,B}$ and $P_{x\geq 2}$ are expressed by the following equations:

(3) $$P_{x=1,B}=\frac{\lambda_{B}\cdot I_{Int}\cdot e^{-\left(\lambda_{B}+\lambda_{S}\right)\cdot I_{Int}}}{\lambda_{B}+\lambda_{S}}=\frac{\lambda_{B}\cdot I_{Int}\cdot e^{-\lambda \cdot I_{Int}}}{\lambda }$$

(4) $$P_{x=1,S}=\frac{\lambda_{S}\cdot I_{Int}\cdot e^{-\left(\lambda_{B}+\lambda_{S}\right)\cdot I_{Int}}}{\lambda_{B}+\lambda_{S}}=\frac{\lambda_{S}\cdot I_{Int}\cdot e^{-\lambda \cdot I_{Int}}}{\lambda }$$

(5) $$P_{x\geq 2}=1-P_{x=0}-P_{x=1}=1-e^{-\lambda \cdot I_{Int}}-\lambda \cdot I_{Int}\cdot e^{-\lambda \cdot I_{Int}}$$

Likewise, $P_{y=1,S}$, $P_{y=1,B}$ and $P_{y\geq 2}$ can also be calculated using similar formulas.

The energy consumed on transmitting the emergency data is calculated next. For upload data, such upload operations include the transmissions without collisions and the transmissions with collisions. Additionally, for download data, the operations for small data, big data or more than one datum are also different. If a collision occurs, the emergency data need to be retransmitted. Hence, the average power spent by a node on transmitting can be written as:

(6) $$\begin{array}{c} P_{Node\_TX}=\left(\frac{P_{x=1}\cdot \left(T_{Data}+T_{TX\_wu}\right)}{n\cdot I_{Int}}\cdot P_{TX}+\frac{\sum_{k=2}^{n}P_{x=k}\cdot 2k\left(T_{Data}+T_{TX\_wu}+T_{CCA}\right)}{n\cdot I_{Int}}\cdot P_{TX}\right)+\left(\frac{P_{y=1}\cdot \left(T_{Ack}+T_{TX\_wu}\right)}{n\cdot I_{Int\_down}}\cdot P_{TX}+\frac{\sum_{k=2}^{n}P_{y=k}\cdot 2k\left(T_{Ack}+T_{TX\_wu}+T_{CCA}\right)}{n\cdot I_{Int\_down}}\cdot P_{TX}\right) \end{array}$$

The first part of Equation (6) represents the energy spent by the nodes to transmit data to the coordinator, and the second part signifies the energy for the nodes to send acknowledgment frames for the download data. The first part includes the energy cost on transmission operations without collision and the energy spent on the transmission operations with collisions. The second part comprises the energy for acknowledging one download datum or more download data. $P_{x=k}$ is the probability that there were *k* upload data transmitted simultaneously, and $P_{y=k}$ is the probability that there were *k* download data. $P_{x=k}$ can be written as:

(7) $$P_{x=k}=\frac{{\left(\lambda \cdot I_{Int}\right)}^{k}}{k!}\cdot e^{-\lambda \cdot I_{Int}}$$

$P_{y=k}$ can be calculated similarly. Next, we calculate the energy spent by the coordinator on receiving operations. During the data section of an upload interposition interval, the coordinator switches to receiving mode to receive the potential emergency data frames from nodes. If there is not a frame, it keeps listening for a while and then abandons listening. Otherwise, it receives the frames and carries out corresponding operations. The average receiving power the coordinator consumes can be expressed as:

(8) $$\begin{array}{c} P_{Coor\_RX}=\frac{2\left(\varepsilon_{\text{TX}}+\varepsilon_{RX}\right)\cdot SI+T_{RX\_wu}+T_{i\_data}+P_{Big\_up}\cdot \left(T_{CAP\_1}-\sum T_{Ack\_1}\right)+P_{x\geq 2}\cdot \left(T_{CAP\_2}-\sum T_{Ack\_2}\right)}{I_{Int\_up}}\cdot P_{RX}+\frac{2\left(\varepsilon_{\text{TX}}+\varepsilon_{RX}\right)\cdot SI+T_{RX\_wu}+T_{i\_ack}+P_{Big\_down}\cdot \left(T_{CAP\_1}-\sum T_{Ack\_1}\right)+P_{y\geq 2}\cdot \left(T_{CAP\_2}-\sum T_{Ack\_2}\right)}{I_{Int\_down}}\cdot P_{RX} \end{array}$$

where $T_{i\_data}$ is the length of the data section of an interposition interval, $P_{Big\_up}$ is the probability that there are big emergency data generated during an upload interposition interval, $T_{CAP\_1}$ and $T_{CAP\_2}$ denote the lengths of CAP1 and CAP2, respectively, and $\sum T_{Ack\_1}$ and $\sum T_{Ack\_2}$ represent the total time spent by acknowledgment frames in CAP1 and CAP2. $T_{i\_ack}$ is the length of the ack section of an interposition interval, and $P_{Big\_down}$ is the probability that there are big emergency data generated during a download interposition interval.

The coordinator needs to broadcast beacon frames periodically and acknowledge the emergency data. In addition, if there are big data or more than one datum, additional beacon frames need to be transmitted. In addition, the coordinator needs to transmit the download data to the nodes. The average transmitting power the coordinator consumes can be expressed as:

(9) $$\begin{array}{c} P_{Coor\_TX}=DC_{BF}+\left(\frac{T_{i\_ack}}{T_{Event\_up}}+\right.\left.\frac{P_{x\geq 1}\cdot T_{i\_ack}+\left(P_{x=1,B}+P_{x\geq 2}\right)\left(T_{Beacon}+T_{RX\_wu}+T_{CCA}\right)}{I_{Int\_up}}\right)P_{TX}+\left(\frac{T_{i\_data}}{T_{Event\_down}}+\right.\left.\frac{P_{y\geq 1}\cdot T_{i\_data}+\left(P_{y=1,B}+P_{y\geq 2}\right)\left(T_{Beacon}+T_{RX\_wu}+T_{CCA}\right)}{I_{Int\_down}}\right)P_{TX} \end{array}$$

Because MEM-MAC uses interposition slots to deliver the emergency data, the data delivery delay of MEM-MAC strongly depends upon the interposition interval. The average time emergency data has to wait before they can be transmitted is IInt2. The time for a node to transmit emergency data to the coordinator is $T_{Data}$. If there is more than one datum produced during an interposition interval, the collisions of these data frames will lead to the extra CAP2 period longer delay being caused. From above, the average delivery delay of MEM-MAC is given by:

(10) $$D_{Data}=\frac{I_{Int\_up}}{2}+T_{Data}+T_{i\_ack}+\frac{P_{x\geq 2}\cdot I_{CAP\_2}}{2(P_{x\geq 2}+P_{x=1})}$$

The lengths of $I_{Int\_up}$ and $I_{CAP\_2}$ are determined during the working process of MEM-MAC. The coordinator records the occurrence times of the data, and it processes the data arrival rates to decide an appropriate value for $I_{Int\_up}$. Let *λ* be the data arrival rate. Experiments show an $I_{Int\_up}$ is appropriate if $\lambda \cdot I_{Int\_up}<1$. As for $I_{CAP\_2}$, the value is estimated by the coordinator according to *λ* and $I_{Int\_up}$. The coordinator generally sets a larger value for the CAP2 period so that it can accommodate all potential frames.

The rapid wake-up mechanism is one feature of MEM-MAC. Both the data section of a download interposition slot and the ack section of an upload interposition slot can be used by the coordinator to wake up all of the nodes. After receiving the wake-up message and a beacon frame, all of the nodes are woken up. The average delay of a wake-up message can be calculated by:

(11) $$D_{Wakeup}=\frac{I_{Int}}{2}+T_{i\_data}+T_{i\_ack}+T_{Beacon}$$

## 5. Performance Evaluation

MEM-MAC is designed to reduce energy consumption, while satisfying the real-time demand of medical emergency frames at the same time. Taking IEEE 802.15.4 as the baseline and adopting OMNeT++ as the simulation tool, the energy efficiency and data delivery delay of MEM-MAC are evaluated in this section. The reason for using 802.15.4 and not using 802.15.6 lies in the following: (1) 802.15.6 is not implemented in OMNeT++ and any other simulation tools. However, 802.15.4 has been implemented in OMNeT++ and many other simulation tools. (2) Although 802.15.6 was specially designed for BSNs and it can cater to many kinds of BSN applications, it does not constitute a complete MAC protocol. In fact, 802.15.6 only outlines the basic elements that are essential to ensure the interoperability among 802.15.6-compliant devices, such as packet formats, message exchange protocols, *etc.* [12]. When 802.15.6 is applied in a practical application, it cannot be used directly. On the contrary, it must be customized and tailored according to the requirements of the application. From this point, 802.15.6 is no more appropriate than 802.15.4 as the baseline and design starting point.

Both the numerical analysis and the simulation results are adopted to evaluate the performance. The numerical analysis of IEEE 802.15.4 is from [23]. By fixing the emergency data occurrence interval and changing the beacon frame interval and interposition slot interval, the energy efficiency and data delivery delay of both protocols and how these performances change with the intervals are first examined. Next, by fixing the beacon frame interval and interposition slot interval and changing the data occurrence frequency, the performances of both protocols are evaluated, and how the performances vary according to the data occurrence interval is examined.

The experiments adopt a star BSN with one coordinator and 20 nodes. It is assumed that the radio parts of all nodes and the coordinator are based on CC2520. The wake-up time of the radio is 0.5 ms. The voltage, receive current and transmit current are set to be 1.8 V, 22.3 mA and 25.8 mA, respectively. The parameters related to the MAC protocol are set according to IEEE 802.15.4. The beacon lengths of IEEE 802.15.4 and MEM-MAC are assumed to be 30 bytes and 34 bytes. The acknowledgment frame length of IEEE 802.15.4 is 5 bytes. For MEM-MAC, the data frame and acknowledgment frame used for the emergency data are 10 bytes and six bytes.

Four MAC schemes are compared when comparing the energy consumption of nodes. The first scheme is IEEE 802.15.4, and the other three are MEM-MAC schemes, which take the same interposition interval and different beacon frame intervals. Let BI be the beacon frame interval and $I_{Int\_up}$ be the upload interposition slot interval, then the number of interposition slots in a superframe can be calculated by NI=BIIInt_up. As a consequence, the three MEM-MAC schemes compared can be distinguished by NI. When comparing the energy consumption of the coordinator, IEEE 802.15.4 schemes with different duty cycles are used. As for the download data, the occurrence interval is set to be identical to the upload data occurrence interval of one node. $I_{Int\_down}$ is set to be $10\cdot I_{Int\_up}$ by inserting one download interposition slot every 10 upload interposition slots.

The performances of IEEE 802.15.4 and MEM-MAC are strongly related to the beacon frame interval of IEEE 802.15.4 and the interposition interval of MEM-MAC. To evaluate the effect of changing these intervals, the average emergency data occurrence interval is fixed to be a quite high value, e.g., 20 min. Such a setting can guarantee the quantity of the generated emergency data to be very little, so that it only has negligible impact on the observation of the effect of changing the intervals. All nodes are set to have the same average emergency data occurrence interval.

**Figure 8:**
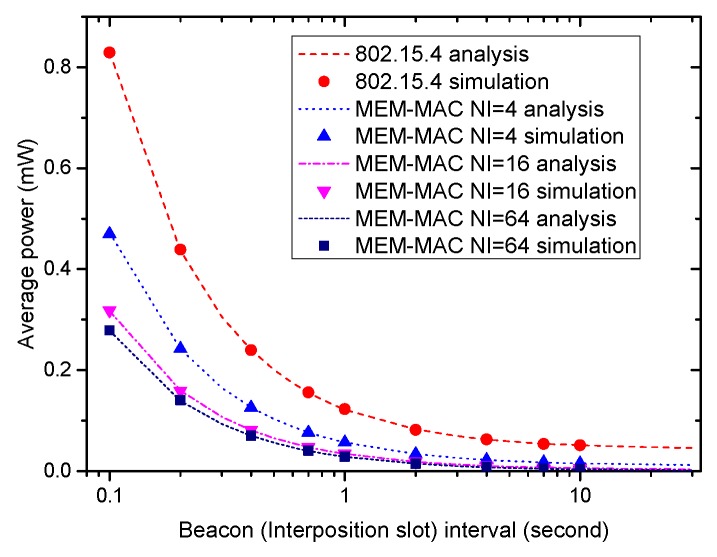
The effects of the beacon frame interval and interposition interval on node average power.

Figure 8 displays how the average power of a node adopting four different MAC schemes changes according to the change of the interposition interval and the beacon frame interval. Owing to the long data occurrence interval set in the experiments, the energy consumption for transmitting the emergency data can be ignored. Under the above settings, the energy is mostly consumed by receiving beacons and the overhearing during the ack section of the interposition slots. Because the numbers of beacons and interposition slots reduce as the interposition interval and the beacon frame interval increase, the average powers of all MAC schemes decrease as the beacon frame interval and interposition interval increase. It also can be seen that all MEM-MAC schemes consume less energy than IEEE 802.15.4, and the average power of an MEM-MAC scheme with a bigger NI value is lower than an MEM-MAC scheme with a smaller NI value. All this can be expounded by the decrease of the transmitting and receiving of beacon frames.

**Figure 9:**
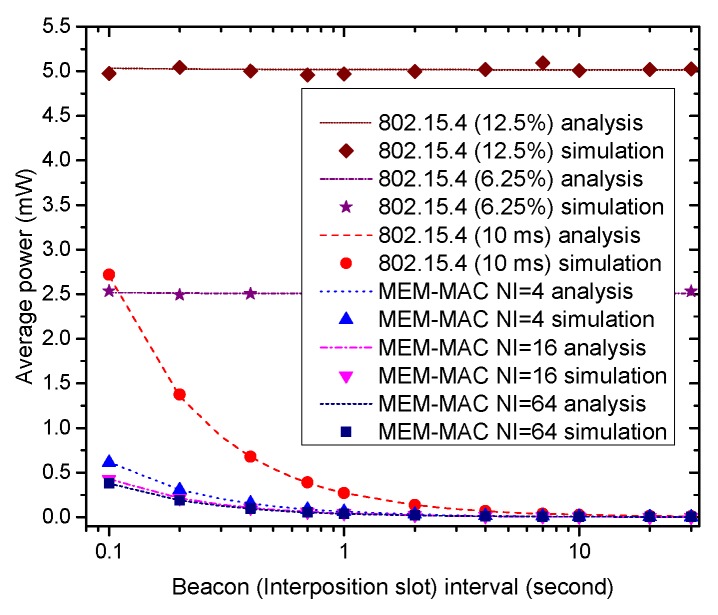
The effects of the beacon frame interval and interposition interval on coordinator average power.

Figure 9 shows how the average power of the coordinator under different MAC schemes changes with the beacon frame interval and the interposition interval. Three different IEEE 802.15.4 schemes are used. The duty-cycles of the first two schemes are 12.5% and 6.25%, respectively. For the third scheme, the length of the active period is a constant value, *i.e.*, 10 ms. It is easy to deduce that the duty cycle of the third scheme decreases with the beacon frame interval increase. It can be observed that the coordinator energy consumption is closely related to the duty cycle. For the first two 802.15.4 schemes, the average power keeps almost the same in terms of the beacon frame interval, because the duty cycles of the two schemes are constant. As for the third IEEE 802.15.4 scheme and three MEM-MAC schemes, the duty cycle decreases as the beacon frame interval or interposition interval increases, and this leads to the decrease of the average powers of these schemes. By eliminating unnecessary beacon frames and using short interposition intervals, three MEM-MAC schemes outscore IEEE 802.15.4 on the coordinator energy consumption.

**Figure 10:**
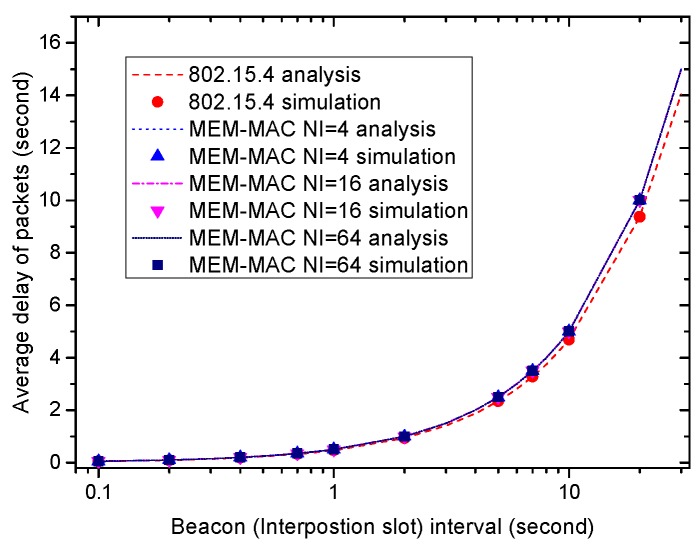
The effects of the beacon frame interval and interposition interval on average frame delay.

Figure 8 and Figure 9 revealed that a longer beacon frame interval and interposition interval can bring higher energy efficiency. Along with the pros, there are always cons. Figure 10 shows the longer data delivery delay brought by the longer beacon frame interval and interposition interval. Just as the figure shows, the data delivery delay grows as the interposition interval and the beacon frame interval increase. This is caused by the longer waiting time incurred by the interval increase. The delivery delay of IEEE 802.15.4 is a little smaller than BI2, and the delays of both MEM-MAC schemes are approximately IInt2. The delivery delay of MEM-MAC is a little larger than IEEE 802.15.4. This is more obvious when the interposition interval and the beacon frame interval take large values. From the figure, to meet the real-time requirements, the beacon frame interval and interposition interval should be set as an appropriate value.

In the following, the effect of the data occurrence interval on the performances is examined based on fixing the beacon frame interval and interposition interval. The average emergency data delivery delay is assumed to be 0.3 s. Based on this, the beacon frame intervals and interposition intervals of all MAC schemes are configured to be 0.5 s according to Figure 10. All nodes are set to have the same average emergency data occurrence interval. 90% of the data is generated to be small data, and 10% of the data is produced as big data. The average data occurrence interval varies from 1 s to 10,000 s, and this signifies that the number of emergency data generated in one interposition interval varies from 10 to 0.001. The setting of the coordinator is set to be the same as the setting of one node.

**Figure 11:**
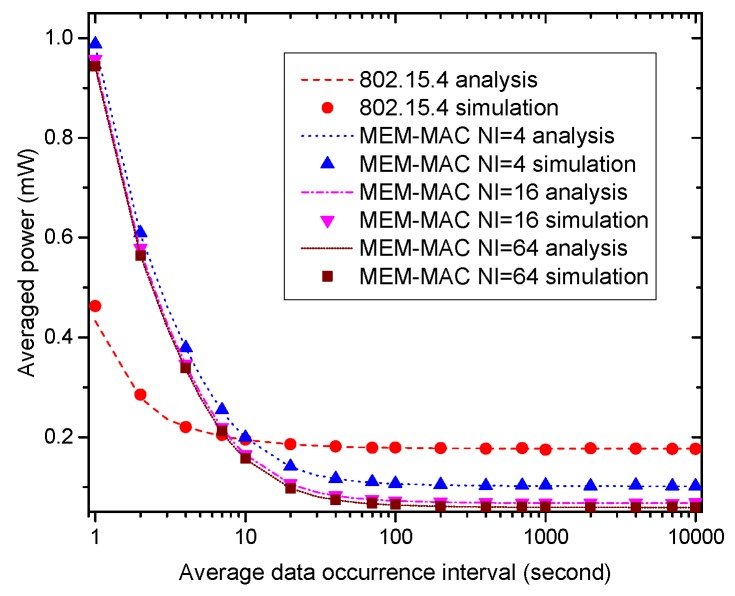
How the node average power changes with the average data occurrence interval.

Figure 11 displays how the averaged power of a node changes with the average data occurrence interval. From the figure, the averaged powers of three MEM-MAC schemes are higher than IEEE 802.15.4 when the data occurrence interval takes a small value, and the energy consumptions of these three schemes decrease rapidly as the data occurrence interval increases. The averaged power of IEEE 802.15.4 only increases a little higher as the data occurrence interval becomes small. The energy consumptions of three MEM-MAC schemes are far higher than IEEE 802.15.4 when the data occurrence interval is small. Yet, the averaged powers of three MEM-MAC schemes quickly become lower than IEEE 802.15.4 as the data occurrence interval increases. This is because when the data occurrence interval becomes small, the emergency data frames cause frequent collisions. These collisions incur frequent CAP2 periods and superframe breaks, which result in frequent frame retransmissions and sending and receiving of extra beacon frames. These operations inevitably cause higher energy consumption.

**Figure 12:**
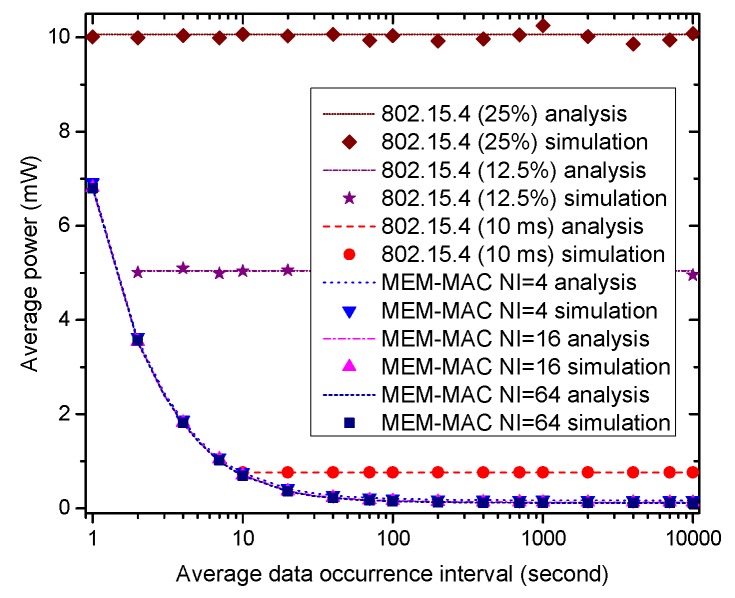
How the coordinator average power changes with the average data occurrence interval.

Figure 12 shows how the average power consumed by the coordinator changes with the average data occurrence interval. For the three IEEE 802.15.4 schemes, the energy consumption keeps constant because the duty cycle keeps constant. For the second and third 802.15.4 schemes, their duty cycles are not enough to transmit the data when the data occurrence interval takes small values, so these parts are not drawn in the figure. As Figure 12 shows, the three MEM-MAC schemes cost high averaged power when the data occurrence interval takes a small value, and the averaged power of the three schemes lowers quickly as the data occurrence interval increases. The averaged power of the three MEM-MAC schemes is higher than the second 802.15.4 scheme, as the data occurrence interval is small; yet, the averaged power of the three MEM-MAC schemes quickly becomes smaller than the third 802.15.4 scheme as this interval grows. The reason to explain the above phenomena also lies in the frequent collisions of data frames and, so, incurring a high duty cycle when the data occurrence interval is small.

**Figure 13:**
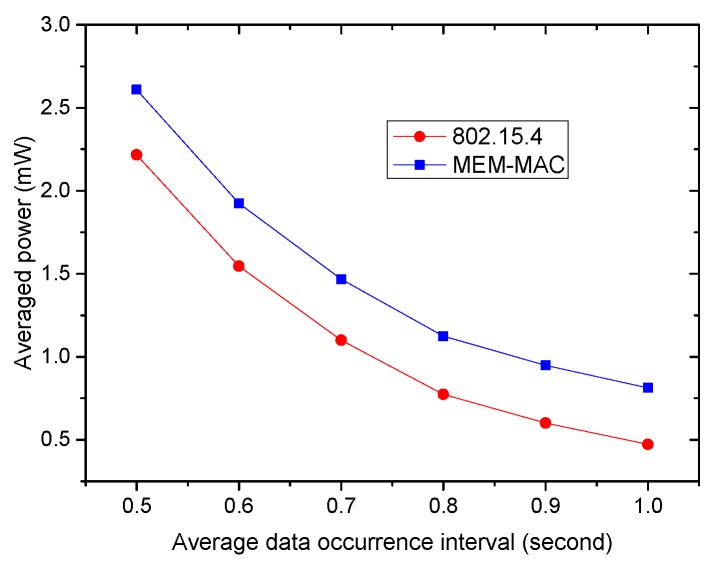
The node energy consumptions when traffic is busy.

From Figure 11, it can be seen that MEM-MAC costs more energy than IEEE802.15.4 when the traffic is busy. To compare the energy efficiencies of MEM-MAC and 802.15.4 when the traffic becomes even busier, more simulations were carried out, and the result is shown in Figure 13. The figure shows that the average power of MEM-MAC is about 0.5 mW higher than 802.15.4 when the traffic is busy. Because the beacons and CAP1 periods of MEM-MAC generally counteract the beacons and CAP periods of 802.15.4, the excessive energy is spent on the interposition slots and extra CAP2 periods. Because 0.5 mW is quite handsome for low power BSN nodes, MEM-MAC should not be used when the traffic is busy.

**Figure 14:**
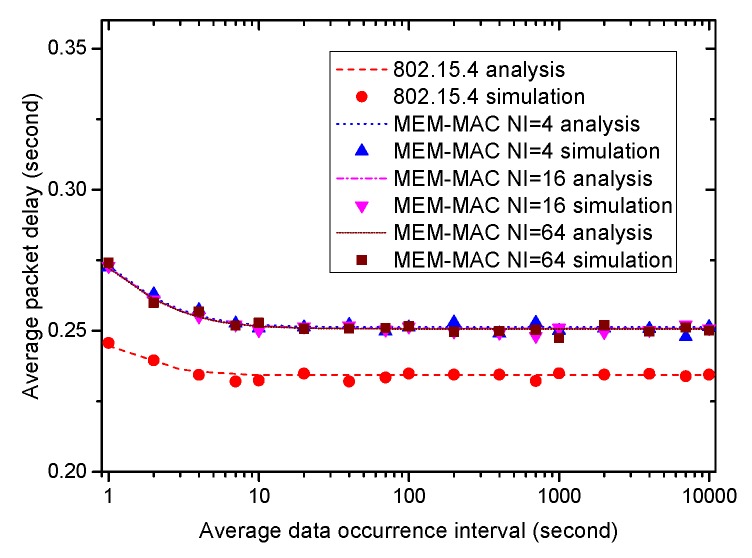
How the average frame delay changes with the average data occurrence interval.

Next, we evaluate how the average frame delay changes the average data occurrence interval. The average frame delays of four MAC schemes are shown and compared in Figure 14. From the figure, all schemes can meet the time delay demand of the emergency data, which is set to be 0.3 s. For IEEE 802.15.4, except for the small increase when data occurrence interval takes small values, the average delay almost remains constant as the average data occurrence interval varies from 1 s to 10,000 s; while for MEM-MAC, the average frame delays of the three schemes all increase when the average data occurrence interval becomes small and lower to a little bigger than 0.25 s with the average data occurrence interval increases. The extra CAP periods and beacon frames are also responsible for these phenomena.

**Figure 15:**
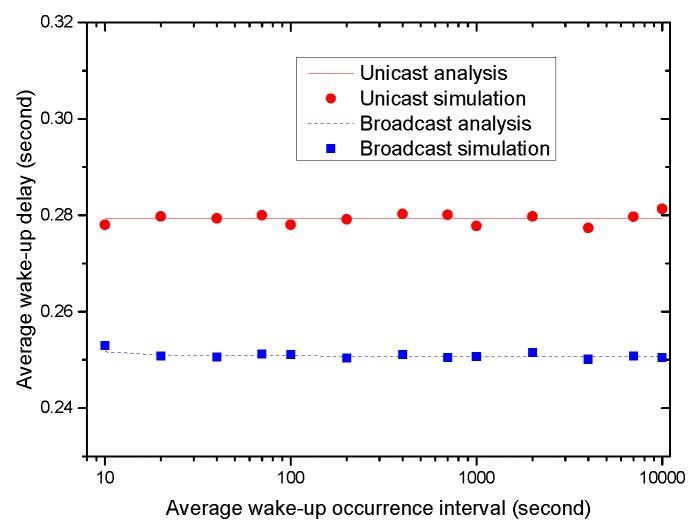
The wake-up delays of two wake-up mechanisms.

Next, we evaluate the time delay and energy efficiency performance of the wake-up mechanism of MEM-MAC. The 802.15.4 standard mentions two specifications about MAC-layer broadcast, but it does not explain what a node should do when it must broadcast a packet to all of its neighbors [25]. Hence, in the experiments, we use MEM-MAC itself as the baseline. We use two mechanisms, unicast and broadcast, to realize the wake-up mechanism. Using unicast, the coordinator sends a wake-up command to each node one by one using a CAP2 period, and each node replies with an acknowledgment frame. While using broadcast, the wake-up command is broadcast to all nodes, and there is no following reply. Figure 15 compares the time delays of two wake-up mechanisms as the wake-up interval increases from 10 s to 10,000 s. The delays stay rather stable as the wake-up interval varies. Additionally, the unicast takes about 30 ms more than the broadcast to wake up all nodes.

**Figure 16:**
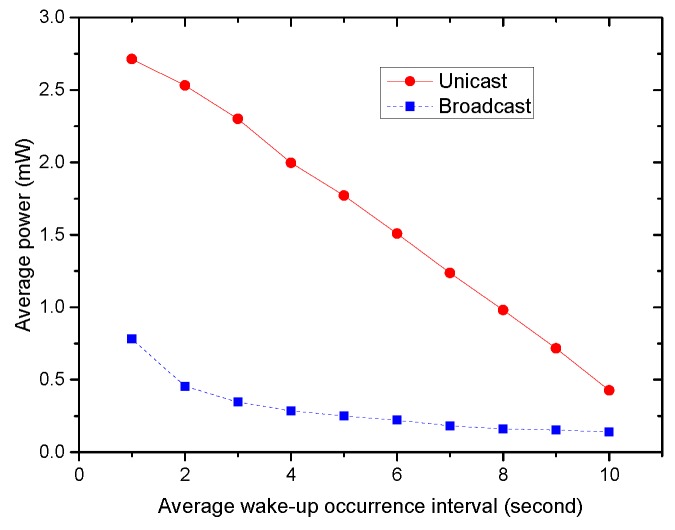
The energy consumption comparison of two wake-up mechanisms.

The broadcast wake-up mechanism outperforms the unicast not only in time delay, but in the energy efficiency. The energy consumption comparison of two wake-up mechanisms is shown in Figure 16. From the figure, the broadcast costs much less energy than the unicast. Although the unicast mechanism has the above disadvantages, it has one notable advantage. It can ensure that every node receives the wake-up command by the acknowledgment mechanism. However, using the broadcast mechanism, it is possible that some nodes are not woken up, because they failed to receive the broadcast wake-up command.

**Figure 17:**
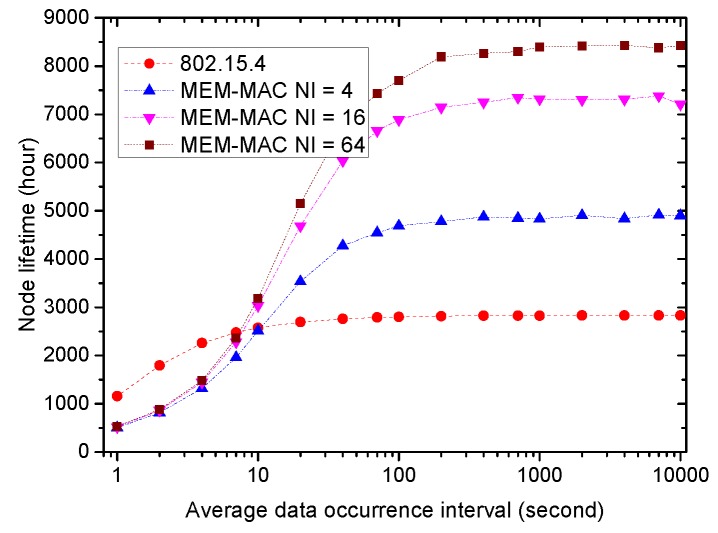
The node lifetime comparison of MEM-MAC and 802.15.4.

Finally, Figure 17 shows the node lifetime comparison of 802.15.4 and MEM-MAC. It is assumed that each node is equipped with one battery with a capacity of 500 mAh, and the beacon interval or interposition interval is set to be 0.5 s. When the traffic is low, it can be observed that one battery can support a node to work for 2800, 4900, 7300 and 8300 h under different MAC schemes. That is to say, a node can last 117, 204, 304 and 345 using different MAC schemes. To extend the node lifetime, the beacon interval or interposition interval should be increased to reduce the duty cycle to a lower level.

From the experimental results, in a medical emergency monitoring BSN where the emergency occurrence frequency is low, MEM-MAC can improve the energy efficiency effectively and meet the time delay demands simultaneously. MEM-MAC is just designed for such BSN applications. However, when the emergency occurrence frequency becomes high, the energy efficiency of MEM-MAC endures a severe degeneration. Therefore, MEM-MAC is not effective for BSN application with heavy traffic. On such occasions, existing MAC protocols optimized for a higher duty cycle, e.g., IEEE 802.15.4 or IEEE 802.15.6, *etc*., can be adopted to accommodate these BSN applications.

## 6. Conclusions

As a MAC protocol specially designed for medical emergency monitoring BSN, a long superframe structure is adopted by MEM-MAC to reduce the transmitting and receiving of unnecessary beacon frames. Short interposition slots are used to deliver the emergency data. To some extent, the interposition slots of MEM-MAC can be regarded as a combination of the beacon frame and CAP period. By reducing the number of beacon frames and adopting short interposition slots, MEM-MAC can achieve high energy efficiency. When the emergency occurrence frequency is not high, MEM-MAC can obtain high energy efficiency and meet the time delay demands simultaneously. However, as the data occurrence frequency grows high, increasing emergency data frames lead to frequent collisions, which, in turn, result in severe degradation of the energy efficiency. Therefore, MEM-MAC is not suitable for BSN applications with high traffic.
